# Liquid biopsy biomarkers for cancer detection, treatment monitoring, and clinical outcome prediction

**DOI:** 10.3389/fcell.2026.1874565

**Published:** 2026-07-20

**Authors:** Akshee Batra, Xena Zheng, Dan Morgenstern-Kaplan, Carey C. Thomson, Gilberto Lopes, Chinmay Jani

**Affiliations:** 1 Department of Medical Oncology, Sylvester Comprehensive Cancer Center, University of Miami, Miami, FL, United States; 2 Department of Medicine, Mount Auburn Hospital, Beth Israel Lahey Health, Cambridge, MA, United States; 3 Department of Medicine, Harvard Medical School, Boston, MA, United States

**Keywords:** circulating tumor cell (CTC), circulating tumor RNA, liquid biopsy, lung cancer, methylation and prognosis

## Abstract

Liquid biopsy now provides minimally invasive access to tumor-derived genomic and epigenetic information across the lung cancer continuum, and its clinical role continues to expand. This review examines that role across cancer detection (screening and diagnosis), treatment monitoring (advanced-disease genotyping, minimal residual disease (MRD) assessment, and resistance profiling at progression), and clinical outcome prediction. Plasma-based genotyping is now well established in advanced non-small cell lung cancer (NSCLC), while circulating tumor DNA (ctDNA)-based MRD detection in the curative-intent setting has accumulated a substantial evidence base over the past 5 years. Cell-free DNA (cfDNA) methylation, fragmentomics, and circulating tumor RNA (ctRNA) are emerging as complementary modalities, particularly when tumor shedding is low. We also consider concordance between liquid and tissue biopsies, the use of cerebrospinal fluid (CSF) ctDNA in central nervous system (CNS)-involved disease, and the practical issues of cost, reimbursement, and access that shape clinical adoption. The current state of the field can be framed across three tiers of evidence, with established applications, applications under prospective evaluation, and applications not yet ready for routine clinical use. No multi-cancer early detection (MCED) test has shown a mortality benefit to date, and ctDNA-guided treatment changes in metastatic disease still lack randomized overall-survival data.

## Introduction

Lung cancer remains the leading cause of cancer-related mortality worldwide and is often diagnosed at advanced stages. Although low-dose computed tomography (LDCT) screening has improved early detection in high-risk populations, important gaps persist in screening, risk stratification, diagnostic precision, and disease monitoring. Treatment selection now hinges on histology, stage, and the identification of actionable genomic alterations, epigenetic signals, and resistance mechanisms (Rolfo et al., 2021; Baca et al., 2023).

**FIGURE 1:**
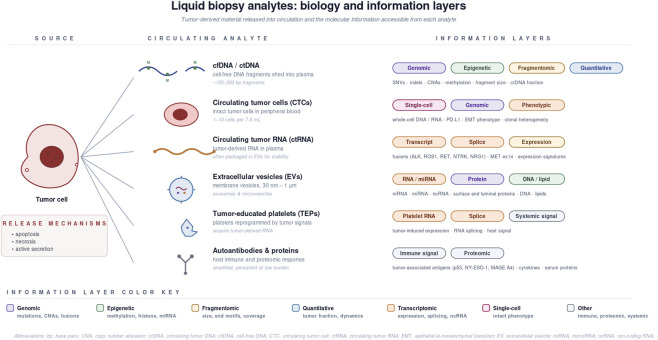
Liquid biopsy analytes, biology and information layers. Schematic of tumor-derived material released into circulation and the molecular information accessible from each analyte. The tumor cell (left) releases material into plasma via apoptosis, necrosis, and active secretion of vesicles, generating six classes of analytes. These include cell-free/circulating tumor DNA (cfDNA/ctDNA), circulating tumor cells (CTCs), circulating tumor RNA (ctRNA), extracellular vesicles (EVs) including exosomes and microvesicles, tumor-educated platelets (TEPs), and tumor-associated autoantibodies and circulating proteins. To the right of each analyte, color-coded pills indicate the principal information layers accessible, including genomic (mutations, copy number alterations, fusions), epigenetic (DNA methylation, histone modifications, microRNAs), fragmentomic (fragment size, end motifs, coverage), quantitative (tumor fraction, dynamics over time), transcriptomic (expression, splicing, non-coding RNAs), single-cell (intact tumor-cell phenotype), and other (immune, proteomic, and systemic signals). Abbreviations: cfDNA, cell-free DNA; ctDNA, circulating tumor DNA; CTCs, circulating tumor cells; ctRNA, circulating tumor RNA; TEPs, tumor-educated platelets.

Tissue and liquid biopsy are increasingly used in an integrative rather than competitive fashion. Tissue retains primacy for histologic confirmation and immunophenotyping, whereas liquid biopsy analyzes tumor-derived material shed into body fluids, most often blood, including ctDNA, circulating tumor cells (CTCs), ctRNA, extracellular vesicles (EVs), and tumor-educated platelets (TEPs), providing dynamic genomic, epigenomic, and transcriptomic insights that complement and extend what tissue can capture (Rolfo et al., 2021; Yin et al., 2020; Roweth and Battinelli, 2021) (Figure 1). Tissue biopsy, although foundational, is invasive, may be unsafe depending on tumor location and patient condition, often yields insufficient material, captures only a single spatial site, and can be difficult to repeat at progression in some patients (Rolfo et al., 2021). Advances in ultrasensitive PCR, next-generation sequencing (NGS), and epigenomic profiling have enabled the integration of liquid biopsy into routine clinical practice. The first plasma-based companion diagnostic, the cobas EGFR Mutation Test v2, was FDA-approved for metastatic NSCLC in 2016, and additional plasma-based assays have since followed (U.S. Food and Drug Administration, 2026).

Although liquid biopsy has historically centered on ctDNA mutation profiling and resistance monitoring, two complementary modalities are becoming increasingly important. ctRNA enhances detection of actionable gene fusions and splice variants that may be missed by DNA-based assays, with combined ctDNA/ctRNA approaches increasing fusion detection yield by approximately 28%–37% compared with ctDNA alone (Giménez-Capitán et al., 2023; Hasegawa et al., 2021; Heeke et al., 2025; Samol et al., 2025; Poh et al., 2025); accordingly, current NCCN NSCLC Guidelines recommend RNA-based testing when DNA-based profiling does not identify a driver oncogene (National Comprehensive Cancer Network, 2026). In parallel, cfDNA epigenetic profiling provides an additional layer of tumor biology through assessment of methylation patterns, fragmentomic features, tumor-suppressor locus hypermethylation, microRNAs, extracellular-vesicle- associated analytes, non-coding RNAs, and histone-modification signatures, capturing chromatin-level information across thousands of genome-wide CpG sites (Vaisvila et al., 2021; Hu et al., 2023; Ramazi et al., 2021). These approaches are particularly informative in low-shedding settings, including screening, early-stage disease, and MRD detection, where mutation-based ctDNA fractions are often very low (Metzenmacher et al., 2022; Wang et al., 2023; Bie et al., 2023).

In NCCN-supported practice, liquid biopsy is most established in advanced or metastatic NSCLC. Common indications include situations where tissue is inaccessible or insufficient for molecular testing (approximately 18%–25% of cases), when invasive biopsy carries unacceptable risk, and for serial detection of resistance alterations and spatial tumor heterogeneity across metastatic sites (Rolfo et al., 2021; National Comprehensive Cancer Network, 2026; Butrynski et al., 2023; Guibert et al., 2020; Marchetti et al., 2025).

This review examines liquid biopsy biomarkers across three core applications in lung cancer: cancer detection (screening and diagnosis), treatment monitoring (including advanced-disease genotyping, MRD assessment, and resistance profiling), and clinical outcome prediction (including recurrence risk and prognostication). For each application, we discuss the roles of genomic, epigenetic, and emerging analytes, as well as the complementary relationship between liquid and tissue biopsy. For this narrative review, PubMed and Embase were searched from January 2015 through April 2026 using terms related to liquid biopsy, ctDNA, cfDNA, CTCs, ctRNA, DNA methylation, fragmentomics, and MRD. Ongoing prospective trials registered on ClinicalTrials.gov, as well as current NCCN, USPSTF, and ESMO guidelines and FDA companion diagnostic listings, were also reviewed. Studies were prioritized based on prospective design, sample size, and lung cancer-specific relevance, while non-English publications and studies focused on other malignancies were excluded.

**FIGURE 2:**
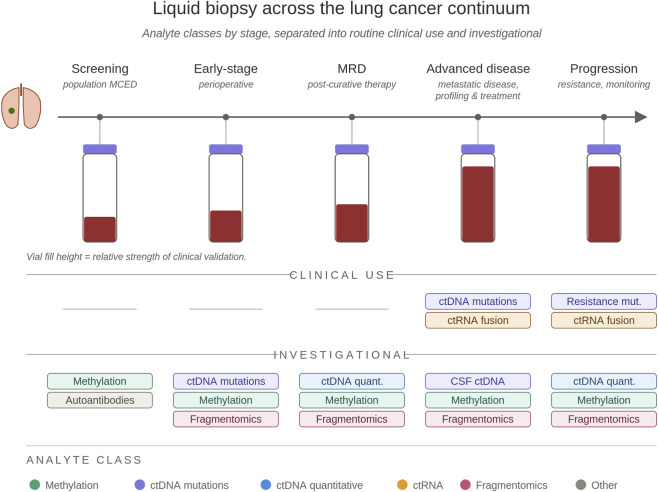
Liquid biopsy across the lung cancer continuum. Plasma-based analyte classes are mapped to five clinical stages of lung cancer: screening, early-stage perioperative care, post-curative MRD monitoring, advanced metastatic disease, and on-therapy progression. Within each stage, analytes are divided into two tiers, those in routine clinical use and those still investigational. Vial fill height shows the relative strength of clinical validation. Only advanced disease and progression currently have liquid biopsy modalities in routine clinical practice. At both stages, ctDNA next-generation sequencing identifies actionable mutations and ctRNA-based assays detect fusions, and both are FDA-approved companion diagnostics for genotyping and resistance assessment. No liquid biopsy modality is yet routine for screening, early-stage disease, or MRD. Cell-free DNA methylation profiling, fragmentomic readouts, autoantibody panels, tumor-informed quantitative ctDNA, and cerebrospinal fluid ctDNA all remain investigational. Color coding matches the analyte class legend. Abbreviations: cfDNA, cell-free DNA; CSF, cerebrospinal fluid; ctDNA, circulating tumor DNA; ctRNA, circulating tumor RNA; MCED, multi-cancer early detection; MRD, minimal residual disease; mut., mutations; quant., quantitative.

Figure 2 maps liquid biopsy modalities to clinical stages across the lung cancer continuum.

Circulating proteins and integrative multi-omic strategies are increasingly being explored in lung cancer alongside established nucleic-acid analytes. Plasma proteomic profiling, increasingly coupled with high-throughput sequencing in multi-analyte frameworks, can complement genomic and epigenomic signals. For example, multi-analyte blood tests that combine circulating protein biomarkers with ctDNA mutation analysis have improved early cancer detection (Cohen et al., 2018), and deep plasma proteomic platforms are now being applied specifically to lung cancer detection and risk stratification (Liang et al., 2025). Looking further ahead, efforts to integrate genomic, transcriptomic, and methylomic data into unified patient-level models, often described as multi-omics digital human avatars, are emerging as a framework for individualized prediction in lung cancer. Such models may ultimately incorporate serial liquid biopsy data alongside clinical and imaging variables (Lococo et al., 2023).

## Lung cancer screening and early detection

LDCT remains the only screening modality with a demonstrated mortality benefit in lung cancer. The National Lung Screening Trial showed a 20% relative reduction in lung cancer mortality with LDCT compared with chest radiography, and the NELSON trial confirmed these findings in a European cohort with lower smoking exposure (de Koning et al., 2020; National Lung Screening Trial Research Team et al., 2011). Based on this, the U.S. Preventive Services Task Force recommends annual LDCT for individuals aged 50–80 years with a ≥20 pack-year smoking history who currently smoke or have quit within the past 15 years (US Preventive Services Task Force et al., 2021). Despite this, uptake remains modest, with 2024 National Health Interview Survey data indicating that only 18.7% of screening-eligible adults in the US report being up to date with LDCT (Bandi et al., 2025). In 2023, the American Cancer Society (ACS) recommendations eliminated the years since quitting requirement for eligibility (Wolf et al., 2024). The NCCN updated their guidelines to align with the ACS in 2025 (Wood et al., 2025). Smoking-based eligibility also misses a substantial proportion of patients, including those who have never smoked, who account for approximately 20% of lung cancer diagnoses in the United States (Chung et al., 2025).

Plasma mutation profiling represents an alternative screening strategy with biological limits in the early-detection context. Localized NSCLC sheds ctDNA at low levels that correlate with tumor size, stage, and histology (higher in non-adenocarcinomas and in solid radiologic patterns), and a further challenge is distinguishing true tumor-derived mutations from clonal hematopoiesis. Machine-learning panels such as Lung-CLiP integrate mutational signatures and fragment-length features to address this challenge (Table 1), but mutation-based approaches remain analytically constrained in the pre-symptomatic, low-volume setting and are unlikely to serve as standalone population screening tools in their current form (Chabon et al., 2020).

**TABLE 1:** Current and emerging screening strategies in lung cancer.

Strategy	Mechanism	Key studies	Sensitivity (early stage)	Specificity	Strengths	Limitations	Status
LDCT (de Koning et al., 2020; National Lung Screening Trial Research Team et al., 2011)	Anatomic imaging of pulmonary nodules	NLST, NELSON (de Koning et al., 2020; National Lung Screening Trial Research Team et al., 2011)	∼70–85% (Early stage) ∼93% (Any stage)	∼73–90%	Only proven mortality benefit; guideline-endorsed	High false-positive rate, radiation; limited compliance (∼19%); excludes those who never smoked	Standard of Care
ctDNA Methylation/MCED (Klein et al., 2021; Schrag et al., 2023)	Targeted methylation sequencing of cfDNA; ML-based cancer signal detection	CCGA, PATHFINDER, PATHFINDER 2, NHS-Galleri (Klein et al., 2021; Nabavizadeh et al., 2025; GRAIL, Inc, 2026; Schrag et al., 2023)	∼17% (Stage I) ∼40% (Stage II)	∼99.5%	High specificity; tissue-of-origin prediction; covers 50+ cancers	Low sensitivity at Stage I; costly; no mortality data yet	Investigational/Commercially available
ctDNA mutational (Chabon et al., 2020)	Plasma NGS for somatic mutations; ML to distinguish from clonal hematopoiesis	Lung-CLiP (Chabon et al., 2020)	∼40% (Stage I) ∼55% (Stage II)	∼95%	Can provide histology-associated signal; tumor shedding data informative	low ctDNA levels at early stage; clonal hematopoiesis interference; histology-dependent shedding	Investigational
Tumor-Aab panel (Sullivan et al., 2021; González et al., 2021)	Detection of host immune response to tumor antigens	ECLS trial (EarlyCDT-Lung), LUSI trial evaluation (Sullivan et al., 2021; González et al., 2021)	∼13–41% (variable by setting)	∼88–91%	Detects immune response that may precede radiographic appearance; inexpensive; non-invasive	Modest sensitivity, especially for small LDCT-detectable tumors; panel-dependent; limited stage-shift data	Investigational Commercially available in limited settings

Abbreviations: AAb, autoantibodies; ctDNA, circulating tumor DNA; LDCT, low-dose computed tomography; MCED, multi-cancer early detection; ML, machine learning.

In contrast, epigenetic profiling of cfDNA, particularly genome-wide DNA methylation analysis, overcomes several of these limitations and represents the most clinically validated blood-based approach for early lung cancer detection. Tumor-specific methylation patterns arise early in oncogenesis and remain detectable even when ctDNA fractions are very low. The Circulating Cell-free Genome Atlas, a prospective multicenter case-control study of over 15,000 participants, demonstrated that whole-genome bisulfite sequencing outperformed mutation-based approaches and informed the development of targeted methylation assays incorporating machine-learning classifiers for cancer detection and tissue-of-origin prediction (Klein et al., 2021). Although early-stage sensitivity remains modest, high specificity is critical in low-prevalence populations to preserve positive predictive value. In lung cancer–specific CCGA analyses, methylation-based approaches outperformed mutation-based classifiers for stage I–II detection, underscoring the central role of epigenetics in blood-based screening. The Galleri test has been evaluated in the PATHFINDER study and NHS-Galleri trial (Nabavizadeh et al., 2025; GRAIL, Inc, 2026; Schrag et al., 2023). In PATHFINDER 2, adding Galleri to USPSTF-recommended screening resulted in more than a seven-fold increase in cancer detection (Nabavizadeh et al., 2025) (Table 1). The NHS-Galleri trial reported a four-fold higher cancer detection rate and a reduction in stage IV diagnoses at interim analysis, although the prespecified primary endpoint of reducing combined stage III–IV cancers was not met (GRAIL, Inc, 2026).

Tumor-associated autoantibody (AAb) panels represent another complementary strategy, leveraging early immune responses to tumor antigens. The EarlyCDT-Lung test is the most extensively studied panel; in the ECLS randomized trial, AAb-positive individuals referred for LDCT demonstrated a stage shift toward earlier diagnosis, although performance in other cohorts has raised concerns regarding sensitivity for small early-stage lesions (Sullivan et al., 2021; González et al., 2021) (Table 1). AAb panels are therefore best viewed as risk-enrichment tools rather than standalone screening modalities. Combinatorial strategies integrating AAb, methylation, and fragmentomic signals are under active investigation.

Overall, blood-based biomarkers are unlikely to replace LDCT but may add value in two complementary roles: upstream risk stratification to refine LDCT eligibility and adjunctive testing alongside LDCT to improve detection of cancers missed by imaging (Table 1). At present, no blood-based screening strategy has demonstrated a mortality benefit, and these approaches remain investigational, requiring prospective validation in diverse real-world populations.

## Molecular diagnostics and treatment selection in advanced NSCLC

The NCCN guidelines recommend comprehensive genomic profiling for all patients with advanced NSCLC, including testing for EGFR, ALK, ROS1, BRAF, KRAS, RET, MET exon 14 skipping, NTRK1/2/3, HER2, NRG1, and PD-L1 (National Comprehensive Cancer Network, 2026). Given the limitations of tissue biopsy, including insufficient material in 15%–30% of cases and challenges with repeat sampling at progression, plasma-based testing is increasingly used as a complementary approach (Lin et al., 2023; García-Pardo et al., 2023).

Plasma ctDNA testing enables rapid detection of actionable alterations, with shorter turnaround times than tissue (median ∼8 vs. 20 days) (Armas et al., 2025). Specificity is high, with >95% concordance for detected mutations, although sensitivity is lower (approximately 70%–80% for mutations and 30%–50% for fusions) (Lin et al., 2023; García-Pardo et al., 2023). In the ACCELERATE trial, a plasma-first strategy reduced median time to treatment initiation from 62 to 39 days (García-Pardo et al., 2023). In EGFR-mutant NSCLC progressing on earlier-generation TKIs, plasma testing facilitates rapid identification of resistance mechanisms, including T790M, MET amplification, ERBB2 amplification, and emergent fusions (National Comprehensive Cancer Network, 2026).

In patients with stage I–III lung cancer, routine use of ctDNA is not advised, and tissue-based molecular testing remains the standard and preferred method for biomarker evaluation. In advanced disease, combined tissue and plasma testing performed concurrently or sequentially may improve the detection of actionable alterations. A positive plasma result identifying a known driver is actionable without tissue confirmation, whereas a negative result should prompt tissue testing to rule out a false-negative plasma assay (National Comprehensive Cancer Network, 2026).

The most established clinical role of liquid biopsy in advanced NSCLC is to accelerate identification of actionable alterations when treatment decisions are time-sensitive. NCCN guidelines support plasma genotyping as complementary to tissue testing, particularly when tissue is limited or delayed (National Comprehensive Cancer Network, 2026). This role is supported by prospective trials. In the NILE study, comprehensive plasma cfDNA genotyping (Guardant360) in 282 untreated patients with metastatic NSCLC was noninferior to physician-selected tissue testing for guideline-recommended biomarkers, identifying at least one biomarker in more patients than tissue alone, with faster turnaround (median 9 vs. 15 days) and high concordance when both were available (Leighl et al., 2019). Similarly, the BFAST trial provided clinical validation of a blood-first strategy, with first-line alectinib in plasma-identified ALK-positive patients achieving high response rates (87.4% by investigator assessment; 92.0% by independent review). Among over 2,200 screened patients, blood-based NGS yielded results in 98.6%, supporting the feasibility of plasma-only screening and demonstrating that liquid biopsy can directly guide targeted therapy selection rather than serve solely as a complementary tool (Dziadziuszko et al., 2021). Building on this, the LIQUIK study further highlights an important evolution in liquid biopsy, demonstrating that while ctDNA alone did not achieve noninferiority to tissue NGS, outcomes based on liquid biopsy-guided therapy were comparable to tissue-based approaches; notably, incorporation of ctRNA alongside ctDNA improved the detection of actionable gene rearrangements and increased overall diagnostic yield, highlighting the clinical value of multi-analyte plasma profiling in capturing alterations less reliably detected by ctDNA alone (Samol et al., 2025).

## Tumor evolution, serial monitoring, and resistance

Emerging “molecular” MRD assays now incorporate methylation and fragmentomic patterns alongside mutation tracking, improving detection sensitivity in low-ctDNA-burden scenarios (Vaisvila et al., 2021; Wang et al., 2023). Tumor-informed assays sequence the patient’s tumor to identify personalized cancer mutations and then design individualized panels that track 16–50 plasma variants (Zhao et al., 2023; Chen and Zhou, 2023). This approach achieves higher specificity (96%–98%) and PPV but requires upfront tumor tissue and a longer development time of 2–4 weeks (Dong et al., 2023; Lu et al., 2025; Boukouris et al., 2025). Tumor-agnostic assays use a predetermined gene panel without prior tumor sequencing, and for that reason are also referred to as fixed-panel assays (Chen et al., 2023a). They enable faster deployment (7–10 days) and can be used when tissue is unavailable. In a recent meta-analysis of 30 NSCLC studies (n = 3,287), landmark MRD sensitivity was similar for tumor-informed and tumor-agnostic assays (42% vs. 44%), as was longitudinal sensitivity (76% vs. 79%). Tumor-informed assays showed higher specificity at both timepoints (landmark 97% vs. 93%; longitudinal 96% vs. 88%) (Lu et al., 2025).

The TRACERx study demonstrated that postoperative ctDNA reliably identifies persistent microscopic disease after resection, particularly in patients who subsequently relapse (Black et al., 2025a) (Table 2). Parallel studies suggest that methylation-based MRD detection can identify recurrence even when mutation-based ctDNA is undetectable (Chen et al., 2023a; Li et al., 2021), highlighting the complementary role of epigenetic signals. Accordingly, newer assays are increasingly integrating methylation-derived features to enhance sensitivity without requiring prior tumor sequencing. Across studies of localized NSCLC, MRD-positive patients have an approximately 6- to 21-fold higher risk of recurrence compared with MRD-negative patients, with a pooled meta-analytic HR ∼6 (95% CI 4.48–8.18) (Schuurbiers et al., 2025; Li et al., 2022; Tan et al., 2024; Zhang C. et al., 2025; Xia et al., 2025).

**TABLE 2:** Summary of key studies evaluating ctDNA-based MRD detection in resected and locally advanced NSCLC.

Study	Design/N/Stage	MRD assay/Panel	Sampling timepoints	Key findings
TRACERx (Abbosh 2023; Black 2025) (Black et al., 2025a; Abbosh et al., 2023; Black et al., 2025b)	Prospective cohort; N = 197→431; Stage I-III	Tumor-informed; WES (200 variants) → WGS/NeXT Personal (1,800 variants)	Preoperative; ≤120 days postoperative; every 3–6 months	Landmark ctDNA+ in 25% of patients (49% of relapses). Ultrasensitive assay: 81% LUAD preoperative (53% stage I). ctDNA clearance associated with improved outcomes
LUNGCA (Xia 2022, 2025) (Xia et al., 2025; Xia et al., 2022)	Prospective multicenter; N = 330/233; Stage I-III	Tumor-agnostic; 769-gene/customized panel	Preoperative; postoperative day 3 and month 1; every 3–6 months × 3 years	MRD + HR 11.1. Post-treatment MRD: PPV 100%, NPV 90.3%. Targetable mutations + TKI: HR 0.01 (p = 0.005)
Qiu 2021/Zhang 2025 (Qiu et al., 2021; Zhang et al., 2025b)	Prospective cohort; N = 261; Stage I-III	Tumor-informed; ultradeep NGS, patient-specific variants	Postoperative; post-ACT; every 3–6 months	Lead time 88 days. Stage II-III MRD + benefited from ACT. Updated: PPV 91.3%, NPV 92.8%, lead time 5.2 months
LUCID (Gale 2022) (Gale et al., 2022)	Prospective; N = 88; Stage I-III	Tumor-informed (RaDaR); WES, 48 patient-specific variants	Pre-treatment; 1–3 days postoperative; 2 weeks-4 months post-treatment	MRD + HR 14.8 (RFS), HR 5.48 (OS). Lead time 212.5 days. Specificity >98.5%
LEMA (Schuurbiers 2025) (Schuurbiers et al., 2025)	Retrospective validation; N = 130; Stage 0-III	Tumor-informed (RaDaR); WES, patient-specific	Pre-treatment; ≥14 days post-treatment; longitudinal	Specificity 97%, PPV 92%, NPV 84%. Combined with LUCID: HR 11.4 (RFS), HR 8.1 (OS). Lead time 204 days
Tan 2024 (Tan et al., 2024)	Retrospective; N = 57; Stage I-III	Tumor-informed (Signatera); WES, 16 patient-specific SNVs	Preoperative; postoperative; longitudinal	Preoperative ctDNA + HR 3.54. Postoperative ctDNA+ → 100% recurrence. Longitudinal HR 16.1. Lead time 2.8 months
Li 2022 (Li et al., 2022)	Prospective; N = 119; Stage I-IIIA	Tumor-agnostic; 425 cancer-related genes	Preoperative; ≤1 month postoperative; every 3–6 months × 3 years	Preoperative HR 2.42 (RFS). Postoperative HR 3.04. Longitudinal HR 3.46 (RFS), HR 9.99 (OS). Lead time 8.7 months
NADIM II MRD (Provencio 2026) (Provencio et al., 2026)	Prospective trial substudy; N = 60; Stage IIIA-IIIB	Tumor-agnostic (Guardant Reveal)	Pre-NAT; postoperative; adjuvant	MRD + HR 10.2 (EFS), HR 10.0 (OS). MRD− at ≥2 timepoints: 100% alive. Prognostic beyond pCR.
Chaudhuri 2017 (CAPP-Seq) (Chaudhuri et al., 2017)	Prospective; N = 40; Stage I-III	Tumor-informed (CAPP-Seq); 128-gene selector	Pre-treatment; post-treatment; longitudinal	MRD detected in 94% of patients with recurrence. Lead time 5.2 months. Specificity 96%. Actionable mutations 53%
PROPHET (Chen 2023) (Chen et al., 2023b)	Prospective; N = 181; Early stage	Tumor-informed (PROPHET); 50 patient-specific variants	Preoperative; postoperative; longitudinal	LOD 0.004%. Lead time 299 days. Proposed TNMB staging outperformed TNM.
Jung 2023 (EGFR-mutant) (Jung et al., 2023)	Prospective; N = 278; Stage I-IIIA	Tumor-informed (ddPCR); EGFR ex19del/L858 R	Preoperative; 4 weeks postoperative; every 6 months × 5 years	MRD+ 3-year DFS 50% vs. 84%. Lead time: 69% (ex19del) vs. 20% (L858 R)
Pan 2023 (CRT, locally advanced) (Pan et al., 2023)	Prospective; N = 139; Locally advanced	Tumor-informed (targeted NGS); patient-specific variants	Baseline; on-RT; after RT; longitudinal	Undetectable MRD (on-RT + after RT) associated with improved survival. Longitudinal MRD−: 2-year csPFS 88.4%
PhasED-Seq (Isbell et al., 2024)	Prospective; N = 46; Early stage	Tumor-informed (PhasED-Seq); WGS, phased variant panels	Postoperative landmark; longitudinal	LOD95 1 ppm. MRD+ → 100% recurrence. Sensitivity 67% vs. 28% (CAPP-Seq). Adjuvant HR 8.2
Rosenlund 2025 (Rosenlund et al., 2025)	Prospective multicenter; N = 45; Stage I-III	Tumor-agnostic (CAPP-Seq)	Pre-treatment; FU1 (0.5–4.5 months); FU2 (4.5–7.5 months)	Post-treatment ctDNA + associated with shorter RFS. FU2 sensitivity 50%. Timing depends on treatment type
MRD-EDGE (Widman 2024) (Widman et al., 2024)	Prospective; multiple cohorts	Tumor-informed (WGS + ML); SNV + CNV enrichment	Preoperative; postoperative; on-treatment	∼300× signal enrichment. CNV detection from 200 Mb aneuploidy. Enables plasma-only monitoring
DART (Horndalsveen et al., 2025)	Prospective phase II; N = 86; Stage III	Tumor-agnostic; 293 genes (hybrid capture)	Baseline; 1 month post-CRT; during durvalumab	ctDNA+ within 4 months post-CRT: HR 4.7 (PFS). Death within 24 months: OR 16.48
MEDAL (Chen 2023) - Methylation (Chen et al., 2023a)	Prospective + validation; N = 195 + 36; surgical	Tumor-informed methylation (timMRD); ultra-deep bisulfite sequencing	Baseline; postoperative	timMRD HR 3.08 (p = 0.002); validated HR 2.80. Outperformed mutation-based ctDNA in stage I. NPV 97.2%

Abbreviations: ACT, adjuvant chemotherapy; CAPP-Seq, Cancer Personalized Profiling by Deep Sequencing; CNV, copy number variation; CRT, chemoradiotherapy; csPFS, cancer-specific progression-free survival; ddPCR, droplet digital PCR; DFS, disease-free survival; EFS, event-free survival; EGFR, epidermal growth factor receptor; ex19del, exon 19 deletion; HR, hazard ratio; LOD, limit of detection; LUAD, lung adenocarcinoma; MRD, minimal residual disease; NGS, next-generation sequencing; NPV, negative predictive value; OS, overall survival; pCR, pathological complete response; PFS, progression-free survival; PPV, positive predictive value; RaDaR, rare and deep sequencing for residual disease; RFS, recurrence-free survival; RT, radiotherapy; SNV, single nucleotide variant; TKI, tyrosine kinase inhibitor; TNMB, tumor-node-metastasis-blood; WES, whole-exome sequencing; WGS, whole-genome sequencing.

Taken together, ctDNA-based MRD assessment represents a powerful tool for early risk stratification and may help identify patients who could benefit from closer surveillance or treatment intensification. In the LUNGCA study, post–comprehensive-treatment MRD status (after surgery plus adjuvant therapy) was a stronger predictor of recurrence than immediate postoperative status (PPV 100% vs. 90%; NPV 90.3% vs. 90.1%). Among MRD-positive patients with targetable alterations, adjuvant TKI therapy improved recurrence-free survival, whereas adjuvant chemotherapy did not (Xia et al., 2025) (Table 2). Ongoing trials are evaluating MRD-guided strategies in resected NSCLC, including treatment intensification for MRD-positive patients and de-escalation for MRD-negative patients (Zhang et al., 2024; AstraZeneca, 2024). The MERMAID-1 trial (NCT04642469) is a phase III study assessing durvalumab versus placebo in ctDNA-positive patients after curative-intent therapy (AstraZeneca, 2024). Similarly, the ADAURA trial MRD analysis (NCT02511106) demonstrated that most DFS/MRD events in the osimertinib arm occurred after treatment cessation (68%) (Herbst et al., 2025), supporting investigation of extended adjuvant therapy durations, as being explored in ADAURA2 (NCT05120349) and TARGET (NCT05526755) (Soo et al., 2024). Additional de-escalation strategies are under evaluation in studies such as CTONG 2201 (NCT05457049) which is prospectively evaluating an adjuvant-free strategy for patients with longitudinally undetectable MRD after radical resection of stage IB–IIIA NSCLC (Zhang et al., 2024). Similarly, the DNA-PREDICT trial is a phase II study evaluating whether ctDNA can predict response to neoadjuvant therapy and guide adjuvant pembrolizumab use or de-escalation strategies in early-stage NSCLC (NCT06902272).

In metastatic disease, serial ctDNA monitoring enables identification of resistance mechanisms and informs treatment sequencing (Takahama et al., 2020). In EGFR-mutant NSCLC, plasma-based detection of acquired T790M using the cobas EGFR Mutation Test v2 has been practice-changing, supported by the AURA3 trial, which demonstrated superior PFS with osimertinib compared with platinum–pemetrexed after prior TKI failure (May et al., 2024; Rotow et al., 2024; Mok et al., 2017). At progression on osimertinib, NGS-based liquid biopsy platforms identify both on-target resistance mutations such as C797S (approximately 13%) and off-target mechanisms including MET amplification and histologic transformation (Rotow et al., 2024; Abbosh et al., 2023; Piotrowska et al., 2026). These insights have therapeutic implications, as demonstrated in the INSIGHT-2 trial, where patients with MET amplification after first-line osimertinib showed encouraging responses to the combination of tepotinib and osimertinib, supporting plasma-based resistance profiling for chemotherapy-sparing strategies (Wu et al., 2024).

Consistent with this, NCCN guidelines for NSCLC recommend parallel ctDNA and tissue-based testing at progression to optimize detection of resistance mechanisms (National Comprehensive Cancer Network, 2026). Although molecular progression may precede radiographic progression, prospective evidence supporting pre-emptive treatment changes based on ctDNA findings alone before radiographic progression remains limited (National Comprehensive Cancer Network, 2026).

Two intrinsic limitations of liquid biopsy warrant emphasis in the resistance setting. First, standard genomic and transcriptomic plasma assays cannot directly identify histologic transformation, such as the conversion of EGFR-mutant adenocarcinoma to a small cell or neuroendocrine phenotype, which is a well-recognized mechanism of acquired resistance under TKI pressure. Although concurrent RB1 and TP53 loss detected in plasma can raise suspicion, tissue re-biopsy remains necessary to confirm transformed histology (Vendrell et al., 2020). Second, plasma analysis cannot currently replace tissue-based assessment of markers required for immunotherapy selection, most notably PD-L1 expression, which is still determined by immunohistochemistry on tissue. Emerging plasma approaches such as cell-free RNA-based PD-L1 quantification remain investigational (National Comprehensive Cancer Network, 2026; Walker et al., 2024).

## Concordance and complementary role of liquid biopsy with tissue biopsy

Concordance between tissue and liquid biopsy in NSCLC is high overall and has improved with successive NGS platforms. A multi-institutional U.S. study of 425 stage III NSCLC cases reported EGFR concordance of 93% (Lin et al., 2023; Malapelle et al., 2026; Sugimoto et al., 2023) (Table 3). Alteration type is a key determinant, with the highest concordance observed for single-nucleotide variants (SNVs) and indels. In the LC-SCRUM-Liquid study, a prospective analysis of 1,062 paired plasma and tissue samples, concordance for common SNV/indel mutations ranged from 72% to 85%, whereas structural rearrangements showed lower concordance with DNA-based assays (Sugimoto et al., 2023). RNA-based NGS improves detection of gene fusions and can identify alterations such as NTRK, NRG1, and MET exon 14 skipping that may be missed by DNA-only panels, supporting its use when no driver alteration is detected (Keller-Evans et al., 2026; Benayed et al., 2019).

**TABLE 3:** Concordance of ctDNA versus tissue biopsy in NSCLC.

Clinical scenario	Concordance/Sensitivity	Clinical significance
Stage III NSCLC (EGFR, all methods) (Sugimoto et al., 2023)	Concordance 93%; sensitivity 45%	Performance improved with contemporary NGS platforms
Stage III NSCLC (NGS 2020–2023) (Sugimoto et al., 2023)	Concordance 96%; sensitivity 53%	Reflects adoption of advanced NGS technologies
Stage III NSCLC (NGS 2017–2019) (Sugimoto et al., 2023)	Concordance 85%; sensitivity 45%	Reduced concordance with earlier-generation platforms
Unresectable NSCLC (National Comprehensive Cancer Network, 2026; Zhang et al., 2019)	Increased concordance and sensitivity compared with resectable disease	Higher ctDNA shedding; liquid biopsy is clinically acceptable
Resectable NSCLC (Qvick et al., 2021; Zhang et al., 2019)	Reduced concordance; stage-dependent (0%–45%)	Limited ctDNA shedding; tissue remains the reference standard
Point mutations (EGFR, KRAS, BRAF) (Keller-Evans et al., 2026; L et al., 2023; Borg et al., 2023)	PPA 77%–85%; pooled sensitivity 69%; specificity 96%–99%	Highest concordance among alteration types; EGFR 78%–82%, KRAS 71%–75%, BRAF 75%–85%
Gene fusions (DNA-based ctDNA) (Sugimoto et al., 2023; Keller-Evans et al., 2026)	PPA 18%–57%	Limited sensitivity for fusion detection; ALK 46%–74%, RET 57%–60%, ROS1 18%–33%
Gene fusions (tissue RNA vs. DNA) (Keller-Evans et al., 2026)	RNA detects 15%–26% more fusions	RNA is essential for comprehensive fusion profiling; combined DNA and RNA comprehensive genomic profiling is recommended
Adenocarcinoma (Qvick et al., 2021; Cai et al., 2021)	Concordance 64%–72% (stage-dependent)	Higher than squamous (64% vs. 47%); concordance increases with stage (0% in stage I to 75% in stage IV)
Advanced/metastatic NSCLC (Chen et al., 2024; L et al., 2023)	Increased concordance and sensitivity; specificity ≥96%	High tumor burden enhances ctDNA yield; liquid biopsy is most reliable in this setting
cfDNA methylation (Borg et al., 2023; Liang et al., 2019; Ezegbogu et al., 2024; Maf et al., 2024; Hsiao et al., 2025)	Pooled sensitivity 47%–62%; specificity 87%–93%	Captures global tumor biology; informative even at low ctDNA fractions

Abbreviations: BRAF, B-Raf proto-oncogene; EGFR, epidermal growth factor receptor; KRAS, kirsten rat sarcoma viral oncogene homolog; NGS, next-generation sequencing; PPA, positive percent agreement; SNV, single nucleotide variant.

Methylation-based cfDNA profiling adds a complementary dimension by capturing genome-wide epigenetic alterations rather than focal sequence changes (Table 4). Tumor-specific methylation signatures identified in tissue are reproducibly detected in plasma, with diagnostic models achieving AUCs above 0.93 in validation cohorts (Machado et al., 2025; Du et al., 2024). Although overall sensitivity remains modest, particularly in early-stage and low-shedding disease (Hu et al., 2023), methylation-based approaches provide additional signal when mutation-based ctDNA is limited, and combined methylation and mutation profiling demonstrates independent prognostic value (Wang et al., 2023; Jiang et al., 2020; Pathak et al., 2025).

**TABLE 4:** Emerging epigenetic and methylation technologies for cfDNA analysis in lung cancer.

Technology	Principle/Input DNA	Key advantages	Key limitations	Lung cancer applications
Whole-genome bisulfite sequencing (WGBS) (Klein et al., 2021)	Chemical conversion of unmethylated C→U; 5–30 ng input	Comprehensive genome-wide coverage; foundational for CCGA/Galleri	DNA degradation; high cost; high input requirement	MCED screening; tissue-of-origin prediction
Enzymatic methyl-seq (EM-seq) (Vaisvila et al., 2021; Olova and Andrews, 2025; Zeng et al., 2026)	Enzymatic (TET2/APOBEC) conversion preserving DNA integrity; 1–10 ng input	Less DNA damage; multimodal (methylation + fragmentation + CNA)	Lower DNA recovery vs. bisulfite (34%–47% vs. 61%–81%)	Early detection; multimodal cancer classification
cfMeDIP-seq (Shen et al., 2018)	Antibody enrichment of methylated fragments; 1–10 ng input	Ultra-low input; genome-wide; tissue-agnostic MRD	Enrichment bias; requires bioinformatic deconvolution	MRD detection; immunotherapy monitoring; pan-cancer detection
MRE-seq (Kwon et al., 2023)	Restriction enzyme digestion at unmethylated CpGs; 5–20 ng input	Detects global hypomethylation; cancer signal origin	Limited to enzyme recognition sites	Cancer detection and tissue-of-origin classification
Targeted methylation panels (Thursby et al., 2026; Maansson et al., 2026)	Amplicon or hybrid-capture of cancer-specific loci; 1–20 ng input	High depth; quantitative; fast turnaround	Limited genomic coverage	Treatment response monitoring; neoadjuvant response prediction
Nanopore sequencing (Katsman et al., 2022)	Direct reading of native methylation marks; variable input	No conversion step; simultaneous base and modification calling	Early development for cfDNA; higher error rates	Investigational; potential for simplified workflows

Abbreviations: APOBEC, apolipoprotein B mRNA, editing enzyme catalytic polypeptide; CCGA, Circulating Cell-Free Genome Atlas; cfDNA, cell-free DNA; cfMeDIP-seq, cell-free methylated DNA, immunoprecipitation sequencing; CNA, copy number alteration; CpG, cytosine-phosphate-guanine; EM-seq, enzymatic methyl sequencing; MCED, multi-cancer early detection; MRE-seq, methylation-sensitive restriction enzyme sequencing; TET2, ten-eleven translocation 2; WGBS, whole-genome bisulfite sequencing.

Disease stage and resectability significantly affect concordance (Keller-Evans et al., 2026; Pathak et al., 2025). Concordance increases progressively with stage, from very low values in stage I to high concordance in stage IV, although the most extreme reported values are not consistently replicated (Qvick et al., 2021; Bontoux et al., 2025) (Table 3). Patients with stage IIIB disease or isolated brain metastases are more likely to have low or undetectable ctDNA due to limited tumor shedding (Normanno et al., 2025; Zhang et al., 2019), and ctDNA testing is therefore not routinely recommended in stages I–III, where tissue remains the standard (National Comprehensive Cancer Network, 2026). Histology also contributes to variability. In early-stage disease, lung squamous cell carcinoma demonstrates higher ctDNA detection rates and tumor methylated fractions than adenocarcinoma (Driussi et al., 2025; Cai et al., 2021), whereas in advanced disease, mutational concordance may be higher in adenocarcinoma, likely reflecting differences in genomic architecture and panel design (Botticelli et al., 2026). Notably, when ctDNA is detectable, concordance can exceed 90% irrespective of histology (Lin et al., 2023; Chen et al., 2024).

Beyond static concordance metrics, prospective data support an integrated diagnostic approach. In the ROME trial, among 1,794 screened patients (400 randomized), those with actionable alterations identified in both tissue and plasma who received matched therapy had improved outcomes compared with standard care (median OS 11.05 vs. 7.70 months; HR 0.74; 95% CI 0.51–1.07; median PFS 4.93 vs. 2.80 months; HR 0.55; 95% CI 0.40–0.76). Outcomes were intermediate when alterations were detected in tissue alone and poorest when identified only in plasma (Marchetti et al., 2025; Botticelli et al., 2026).

Taken together, these findings support an integrative rather than competitive diagnostic strategy. High concordance reinforces confidence in clinical decision-making, while discordant findings, when interpreted in appropriate clinical context, may still provide actionable insights (Rolfo et al., 2021; Lin et al., 2023; Keller-Evans et al., 2026; Chen et al., 2024).

## Cost, reimbursement, and access

In the United States, three liquid biopsy assays (cobas EGFR Mutation Test v2, FoundationOne Liquid CDx, and Guardant360 CDx) have been approved by the FDA as companion diagnostics (U.S. Food and Drug Administration, 2026). This regulatory framework has helped establish a clearer reimbursement pathway for plasma-based genomic profiling when used to identify actionable alterations and guide targeted therapy in advanced NSCLC. Consistent with this, Medicare’s CMS NCD 90.2 provides broad Medicare coverage for FDA-approved or cleared CDx NGS assays in patients with advanced cancer when used to inform treatment decisions, thereby supporting reimbursement for liquid biopsy in this setting (Centers for Medicare and Medicaid Services, 2020).

By contrast, coverage for MRD testing, surveillance, and screening remains more limited and fragmented. No national coverage determination (NCD) exists for liquid biopsy MRD or surveillance testing, and Medicare coverage is instead largely determined through local coverage determinations (LCDs), particularly the MolDX framework, including LCD L38835 (Centers for Medicare and Medicaid Services, 2021). This policy provides limited coverage for minimally invasive MRD assays, including ctDNA-based liquid biopsy tests, but only for patients with a personal history of cancer and only when the assay meets defined criteria for clinical validity and utility. Coverage requires evidence that the assay can detect molecular recurrence before clinical, laboratory, or radiographic recurrence, and that identification of recurrence or progression would lead to a guideline-supported change in patient management. MolDX coverage has been reported for Signatera (Natera, Inc, 2025) in stage I–III NSCLC surveillance and for immunotherapy monitoring, but this represents assay- and indication-specific coverage rather than a broad endorsement of liquid biopsy MRD across lung cancer care. Screening is the most constrained access setting, as liquid biopsy screening tests lack an NCD or LCD pathway, so early-detection applications remain largely investigational or available only through clinical trials, research protocols, or self-pay or commercial channels.

Commercial payer coverage adds another layer of variability. In a 2023 review of private payer and Medicare coverage policies for ctDNA testing, Douglas et al. found that 89% of private policies (Douglas et al., 2023) specified coverage of ctDNA testing for at least one indication. Among 57 private payer policies, 69% covered ctDNA testing for initial treatment selection, whereas coverage was less consistent for progression and MRD. Only 28% of policies addressing progression provided coverage, while 65% of policies addressing MRD provided coverage (Douglas et al., 2023).

Globally, access to liquid biopsy remains uneven and is closely tied to broader reimbursement challenges facing advanced genomic testing. In the 2023 ESMO survey of biomolecular technology access (Bayle et al., 2023) across 48 European countries, liquid biopsy was grouped among the more advanced technologies with generally low availability, often limited to clinical trials or research programs in high-income countries and frequently unavailable in low- and middle-income countries. Lung cancer was an important exception, reflecting the more established role of plasma-based genotyping in advanced NSCLC; even within lung cancer, however, access remains concentrated around treatment selection rather than broader applications such as MRD detection, surveillance, or screening. Cost is a major constraint. While prices for single-gene techniques generally ranged from a few tens to a few hundred euros, more complex NGS approaches had much wider price ranges, reaching up to €3,000 per patient, and the proportion of out-of- pocket expenses increased as testing became more advanced. Reimbursement was the leading barrier to multigene testing, cited more often than for single-gene testing (59% versus 24%) (Bayle et al., 2023).

## Current challenges and future directions

Liquid biopsy in the United States continues to face important biological, technical, and regulatory challenges that limit broader application in early detection and post-treatment decision-making. A key biological constraint is the “shedding limit” for small tumors (Deveson et al., 2021; Avanzini et al., 2020). Most FDA-approved broad-panel assays reliably detect variants at ≥0.5% allele frequency, with some achieving ∼0.1% under optimal conditions (Northcott et al., 2024). Newer ultrasensitive technologies such as PhasED-Seq and NeXT Personal achieve detection in the parts-per-million range (∼0.0001%), and tumor-informed mPCR assays such as Signatera report detection down to ∼0.01% (Northcott et al., 2024; Verma et al., 2020; Reinert et al., 2019). Despite these advances, early-stage detection remains constrained by very low tumor fraction.

Pre-analytical and analytical variables also affect test performance. CAP/CLIA standards govern laboratory workflows, and sample handling is critical (for example, Streck BCT tubes preserve cfDNA stability for up to 5–7 days at room temperature) (Medina et al., 2016). Clonal hematopoiesis of indeterminate potential (CHIP) remains a major confounder, generating false-positive mutation signals and present in approximately 10%–15% of individuals over age 70 (Razavi et al., 2019). Bioinformatic filtering and paired white blood cell sequencing mitigate this in mutation-based assays, while methylation-based assays are inherently less susceptible to CHIP-related interference (Razavi et al., 2019). Turnaround time also constrains clinical utility, as tumor-informed assays offer high specificity but require 2–4 weeks for personalized panel design, whereas tumor-agnostic and methylation-based platforms enable faster results with tradeoffs in sensitivity (Lu et al., 2025). Regulatory pathways are evolving, including FDA consideration of MRD as a surrogate endpoint, which may accelerate adoption.

Artificial intelligence and machine learning are increasingly used to integrate genomic, epigenomic, and fragmentomic data. Meta-analyses of independent validation datasets report pooled sensitivities of ∼78–90% and specificities of 94%–99% for early-stage cancer detection using ML-based models (Cohen et al., 2018; Filis et al., 2026). Combining these data streams will be central to comprehensive tumor characterization from a single blood sample.

The use of ctDNA in CSF is also changing how CNS involvement is evaluated. Because the blood-brain barrier restricts the passage of tumor DNA into the bloodstream, standard blood-based liquid biopsies often miss brain metastases or leptomeningeal disease (LMD). Sampling CSF directly bypasses this barrier. A systematic review and meta-analysis of 26 studies reported a pooled CSF ctDNA detection rate of 86% (95% CI 79%–91%), significantly higher than CSF cytology at 60% (95% CI 36%–81%). Pooled sensitivity was 91.8% and specificity 93.5% for detecting CNS metastases in NSCLC, although individual study detection rates ranged from 82% to 100% (Olayode et al., 2025).

Clinicians increasingly use CSF ctDNA to evaluate CNS metastases including leptomeningeal disease, to monitor disease progression in the brain, and to detect resistance mechanisms such as EGFR T790M that may be invisible in plasma (Zheng et al., 2025). A landmark study of 584 NSCLC patients with CNS metastases found that CSF ctDNA positivity was independently associated with shorter survival (HR 1.9, 95% CI 1.56–2.39; P < 0.0001), and patients with a driver alteration detected by CSF ctDNA derived a survival benefit from CSF-matched therapy (HR 0.78, 95% CI 0.65–0.92; P = 0.003) (Zheng et al., 2025). CSF testing has also shown that the genomic profile of brain metastases can differ from extracranial disease, with more driver alterations, more copy-number variations, and distinct resistance mechanisms (Sun et al., 2025; Brastianos et al., 2015). The current NCCN CNS Cancers Guidelines (v3.2025) note that “when available, assessment of CSF-tDNA increases sensitivity of tumor cell detection and assessment of response to treatment” (National Comprehensive Cancer Network, 2025). CSF ctDNA is not yet FDA-approved as a formal CDx, but institutions are increasingly incorporating these protocols into clinical practice as part of precision neuro-oncology in lung cancer (Seoane et al., 2019).

A further barrier to cross-study comparison is the lack of harmonization across commercial assays and research pipelines. Differences in gene panel content and breadth of coverage mean that the same plasma sample may yield different actionable findings depending on the assay used, which constrains the interpretation of tissue and plasma concordance studies (Deveson et al., 2021; Stetson et al., 2019). Analytical performance is itself heterogeneous. Multi-site benchmarking has shown that above approximately 0.5% VAF mutations are detected reproducibly across platforms, but below this threshold sensitivity diverges widely between assays, and technical rather than biological factors are a major source of inter-assay discordance (Deveson et al., 2021; Stetson et al., 2019). Methodological heterogeneity in bioinformatics compounds this problem, because variability in variant-calling algorithms, filtering thresholds, and quality-control metrics makes direct comparison of datasets generated by different pipelines unreliable in the absence of shared reference standards and reporting frameworks (Deveson et al., 2021).

Related to this is the absence of standardized metrics for the absolute tumor fraction of circulating DNA, a key factor for correct interpretation of results, including the meaning of a negative test. Variant allele frequency (VAF) is frequently used as a surrogate but does not equate to absolute tumor fraction, because it is influenced by copy-number state, clonality, and clonal hematopoiesis. Orthogonal genomic approaches such as genome-wide copy-number-based tumor-fraction estimation (for example, ichorCNA) and epigenomic, methylation-based quantification provide assay-independent estimates of circulating tumor content (Adalsteinsson et al., 2017; Ye et al., 2025). Robust tumor-fraction estimation is the quantitative keystone for interpreting ctDNA clearance dynamics in longitudinal monitoring and for distinguishing a true negative from a low tumor-fraction sample that falls below the assay limit of detection (Adalsteinsson et al., 2017; Ye et al., 2025).

## Conclusion

Liquid biopsy has matured into a clinically deployed tool across the lung cancer continuum, with evidence strength varying considerably by application. The field can be framed across three tiers, including established applications, applications under prospective evaluation, and applications not yet ready for routine use. At the established end, plasma genomic profiling for actionable alterations in advanced NSCLC is the only application supported by FDA-approved companion diagnostics, NCCN guideline endorsement, and randomized clinical utility data. It accelerates time to first-line targeted therapy, identifies acquired resistance and supports treatment sequencing through detection of bypass alterations with broad reimbursement through CMS NCD 90.2.

A second tier, including ctDNA-based MRD detection after curative-intent therapy, is the most actively studied application beyond advanced disease. Tumor-informed assays achieve high specificity in established cohorts, while methylation- and fragmentomics-based multimodal assays are extending sensitivity into low-shedding settings; phase III MRD-guided trials evaluating treatment escalation and de-escalation have yet to report. CSF ctDNA for CNS-restricted disease, ctRNA for fusion detection, and methylation-based tissue-of-origin classifiers sit in this same emerging-evidence tier.

Several other applications are not ready for routine practice. Multi-cancer early detection has shown analytical feasibility but no mortality benefit. The NHS-Galleri trial reported increased early-stage detection but missed its primary endpoint of reducing combined stage III–IV cancers. ctDNA-guided treatment changes in metastatic disease lack randomized OS data, and tumor-associated autoantibody panels and fragmentomics-only screening assays remain investigational and largely uncovered by payers.

The next phase of liquid biopsy in lung cancer will be defined less by new analytes than by disciplined integration of existing modalities (genomics, methylation, fragmentomics, and ctRNA), the completion of randomized MRD and screening trials, and reimbursement frameworks aligned with the evidence. Until then, the principal value of liquid biopsy lies where the evidence is strongest, in advanced-NSCLC molecular profiling and treatment selection, while emerging roles in early detection and MRD await trial readouts and regulatory pathways that will determine whether they become standard care.
